# Exploring locally developed ELT materials in the context of curriculum-based value education in China: challenges and solutions

**DOI:** 10.3389/fpsyg.2023.1191420

**Published:** 2023-10-13

**Authors:** Shiping Deng, Xuemei Wang

**Affiliations:** ^1^Department of Applied Foreign Language Studies, Nanjing University, Nanjing, China; ^2^School of English Studies, Shanghai International Studies University, Shanghai, China

**Keywords:** ELT, locally developed materials, material use, challenges, solutions

## Abstract

The development of locally developed English language teaching (ELT) materials is believed to be conducive to meeting the requirements of local language education policies and practices, and to enhance learners’ agency. In China, in order to implement national educational policy of curriculum-based value education, many locally developed ELT materials have been recently developed. However, there is a paucity of empirical research concerning the actual effects of such materials in practice. This study presents an empirical study of a recently developed set of teaching materials in the context of curriculum-based value education by adopting an approach consisting of questionnaires and interviews to examine the attitudes and experiences of users of these materials. The findings revealed a range of challenges in the use of these materials, including issues related to language and content of the materials, the integration of ideological and value elements, and the development of multi-dimensional teaching materials. The article then proposes possible solutions from the perspectives of optimizing the writing community, carefully selecting teaching materials, innovating the integration of ideological and political elements, and improving the development of multi-dimensional teaching materials. A framework for the evaluation of locally developed ELT materials is also proposed, with the intention of offering a useful benchmark for enhancing the development of such materials.

## 1. Introduction

Teaching materials serve as a significant medium of education and are considered as a universal element in EFL (English as a foreign language) teaching and learning (Hutchinson and Torres, 1994), and as such, are directly related to the direction and quality of talent cultivation. Using the so-called “international” materials(López Barrios and Villanueva de Debat, 2014) that are developed by native speakers in English language teaching (ELT) can undoubtedly amplify students’ immersion in foreign language learning and deepen their understanding of the social and cultural context underlying the language. However, this does not mean that there is no need of locally developed materials. Despite teachers’ efforts to adapt “international” materials, the specific needs of learners in terms of cultural and linguistic differences cannot be addressed. Moreover, it is revealed that neoliberal discourses and values demonstrated in “international” ELT materials outweigh those in locally developed ones (Taki, 2008; Daghigh and Rahim, 2021), which may have negative impacts on students’ values. Language and values are often inseparable (Kramsch, 2015). Material developers need to distinguish and differentiate the specific cultural values carried by the materials they choose, while also strengthening the presentation of local culture, local practices, and local values in ELT materials. Currently in China, material developers are actively attempting to design locally developed ELT materials to promote curriculum-based value education, a national educational policy aimed at incorporating the thoughts of socialism with Chinese characteristics, the core socialist values, and the excellent traditional Chinese culture into all courses in higher education. Studies have investigated how Chinese culture and other cultures (e.g., Anglo-American culture) are represented in locally developed ELT textbooks in China (Liu et al., 2022; Zhang et al., 2022). Nevertheless, there is a paucity of empirical research concerning the actual effects of the locally developed materials in practice, and teachers’ and students’ experiences in using these materials are neglected. Therefore, taking a series of locally developed ELT materials as an example, this study scrutinizes the predominant challenges involved in developing local ELT materials in China in the context of implementing curriculum-based value education. Specifically, the voices of material users, namely teachers and students, are considered in the examination of these issues. Additionally, this study recommends possible solutions for the future development of such materials.

## 2. Literature review

López Barrios and Villanueva de Debat (2014) classifies textbooks into three categories: global textbooks, which are designed for learners of a particular age and level and used worldwide; localized textbooks, which are adapted from global textbooks to a national curricula and a specific context; and locally developed textbooks, which are tailored to a particular region and take into account learners’ backgrounds. What is investigated and discussed in this study belongs to the third type. Garton and Graves (2014) believe that locally developed ELT materials may enable students to express their ideas based on their own cultural and personal experiences.

Many researchers have focused on the culture presented in locally developed ELT materials. Some researchers examine the western culture and beliefs (such as the neoliberal discourses) in local ELT materials (Forman, 2014; Daghigh and Rahim, 2021). Forman (2014), for example, uncovers that cultural assumptions or discourses in ELT materials can be misleading and cause discomfort among teachers. There are also researchers who focus on the local culture in locally developed ELT materials. For example, Toledo-Sandoval (2020) discovers that ELT materials for 5th and 6th graders in Chile concentrate on cultural elements related to “national geography” and “stereotypes and national identity”; however, there is also local cultural elements that may have negative effects on students’ values in those materials.

Previous research results indicate that local material developers have many options at their disposal in the development process. For instance, by investigating three locally developed textbooks that are used in the schools of Chile, Glas (2013) finds that all of these books incorporated cultural representations that are associated with both the United Kingdom and Chile, but each of the three materials adopt a distinct approach to culture, respectively.

Studies also reveal that different stakeholders ought to be included in the development of local materials. In a study by Augusto-Navarro (2015), the author explores the development of local teaching materials for Brazilian schools. The research focuses on how undergraduate and postgraduate students in a teacher training program developed the materials and found that engaging student teachers in the efforts of designing context-specific materials could help them feel empowered and contribute to their professional growth.

Many studies have discussed the material evaluation. The most frequently mentioned individuals are experts, teachers, and students. For example, Khodabakhshi (2014) examines how teachers view a series of materials in terms of its practical, pedagogical, and external aspects by data collected with a questionnaire. Taking on the perspective of experts, Dang and Seals (2018) focus on four primary aspects reflected in Vietnam’s primary ELT materials, including the teaching methodology, use of bilingualism, language differences, and intercultural communication. Students’ voices are also included in ELT material evaluation; for instance, Melissa (2018) analyses students’ reliance on, and critical evaluation and skillful use of ELT materials and the lingua-cultural resources. There are also investigations from multiple perspectives. For example, in a study conducted by Li and Cui (2021), both teachers’ and students’ voices have received the authors’ attention. However, only a few studies focus on how to evaluate locally developed materials. Kirkgoz (2011) investigates local ELT materials used in Turkish Primary Education and identifies two shortcomings of materials in complexity and learnability from the perspectives of teachers and students. Based on the model by Nation and Macalister (2010) and Biria and Boshrabadi (2015) propose a multi-aspectual (principle-driven) approach for the evaluation of local ELT materials used in Iran and identify the problems of those local materials by investigating classroom practices. Methods adopted in these studies include interviewing teachers and students, and administering questionnaire surveys to collect large scale data. It can be seen that locally developed ELT materials have received little attention. In the context of implementing curriculum-based moral education, many local ELT materials have recently been developed and used in Chinese universities and colleges. Chinese researchers proposed some principles that need to be followed when developing local ELT materials in the context of curriculum-based value education (Sun, 2020; Xiao and Zhao, 2023), while others have employed textual analysis methods to analyze the presentation of Chinese culture in such materials (e.g., Zhang and Yu, 2020). Nevertheless, there has been limited research on the material users’ perceptions and feelings of these local ELT materials. Therefore, this study aims to examine the challenges these materials confronted in practice from the perspectives of their users, explore corresponding solutions, and develop an evaluation framework for locally developed ELT materials based on the findings. This framework can serve as a reference for local policy makers, researchers, experts, and teachers, and offer insights for the development of local ELT materials in other regions of the world.

## 3. Methodology

### 3.1. Research aim

This study seeks to explore the challenges faced by users of locally developed English Language Teaching (ELT) materials and to identify potential strategies for enhancing the effectiveness of such materials. By examining the experiences and perspectives of material users, this research aims to contribute to the development of a comprehensive framework for locally developed ELT materials. This framework will be grounded in the insights gained from the study, offering practical solutions to address challenges and maximize the utility of these materials in educational contexts. The research endeavors to provide valuable guidance for educators and curriculum developers in their efforts to design and implement ELT materials that align with the goals of curriculum-based value education, ultimately benefiting English language learners and the broader educational landscape.

### 3.2. Site

This research was conducted at a distinguished provincial university in China, strategically chosen as the research site due to its notable academic standing and its role as a participant in China’s “Double First-Class” university initiative. Additionally, it serves as an exemplary institution for the Ministry of Education’s university English teaching reform efforts, making its College English education program representative within the Chinese higher education landscape.

At the heart of this research lies the implementation of “curriculum-based value education,” a pivotal national educational policy. This policy aspires to infuse Chinese cultural attributes, core socialist values, and traditional Chinese heritage into all facets of higher education. It aims to cultivate not only academic prowess but also moral and intellectual capabilities among students, with the ultimate goal of contributing to China’s ongoing development and construction efforts.

One noteworthy facet of this policy is its universal application, spanning all courses offered by the university, including English Language Teaching (ELT) courses. During the time of this study, the university was actively incorporating curriculum-based value education into its College English course, a program catering to non-foreign-language majors. These selected classes utilized a locally developed ELT material, comprising three volumes, each featuring an integrated coursebook accompanied by a corresponding teacher’s guide.

The integrated coursebooks, predominantly used by students during their English classes, are distinctively designed to reflect local culture, encompassing elements such as the core socialist values and traditional Chinese cultural nuances. A noteworthy feature highlighted by the developers in the preface of these materials is the deliberate incorporation of various moral education elements into the discourse, subtly infusing students’ learning experiences with underlying moral, ideological values, and cultural connotations.

This research, undertaken at this prominent provincial university, not only aligns with national educational policies but also offers a unique vantage point to investigate the development and implementation of teaching materials consistent with these values. It delves into how curriculum-based value education is translated into English language teaching, thereby contributing to a broader understanding of the policy’s implications within the realm of higher education in China. The selected institution’s stature as a “Double First-Class” university and its pioneering role in university English teaching reform make it an ideal location for such an exploration, representative of the changes occurring in Chinese higher education.

### 3.3. Methods

The use of a combination of interviews and questionnaire surveys in this research was deliberate and aimed at providing a comprehensive understanding of the challenges associated with locally developed ELT materials, as well as the perspectives and attitudes of material users, including both teachers and students.

Interviews played a pivotal role in delving deeper into the attitudes and perspectives of material users. These qualitative interviews allowed for a nuanced exploration of the reasons ELT material users’ attitude and provided a platform for participants to elaborate on their experiences and insights. Through interviews, we gained a rich understanding of the challenges faced and the underlying factors contributing to these challenges.

The questionnaire served as a valuable tool for gathering quantitative data to gauge the overall sentiment and experiences of material users. Its design was meticulously crafted to elicit specific information regarding language quality, content appropriateness, the integration of local elements, and multi-dimensional aspects of the materials. The purpose of the questionnaire was to obtain a broad and systematic overview of the challenges faced in using these materials, providing a quantitative foundation for the subsequent qualitative interviews.

One of the primary advantages of employing a questionnaire was its ability to collect data from a relatively large sample, allowing for a broader perspective and general trends to emerge. This quantitative data served as a valuable initial step in identifying key challenges and areas of concern related to the locally developed ELT materials. Additionally, the questionnaire’s structured format enabled the systematic collection of information, ensuring that specific aspects of language quality, content, and integration were systematically evaluated.

The combined use of interviews and questionnaire surveys provided a balanced approach, combining quantitative data for a broader overview with qualitative insights for a deeper exploration of the research topic. The interviews captured the individual experiences and perspectives of material users with depth. The questionnaire helped identify trends and common challenges. This dual-method approach allowed for a more comprehensive analysis, ensuring that the challenges and potential solutions identified in this research are grounded in both quantitative data and qualitative insights.

Overall, the use of questionnaires and interviews was strategic in addressing the research questions comprehensively, blending the advantages of quantitative and qualitative data collection methods to provide a holistic understanding of the challenges and potential solutions associated with locally developed ELT materials.

### 3.4. Data collection and analysis

The author adopted a micro perspective to collect genuine feedback from teachers and students, i.e., the users of the locally developed materials in the context of curriculum-based value education. The selection of participants for this research was guided by several key considerations that align with the research objectives and the specific context of the study. The participants included both teachers and students who were involved in the use and evaluation of locally developed ELT materials.

In choosing these participants, the primary aim was to ensure that they possessed direct and first-hand experience in the locally developed materials. This criterion was crucial to gaining insights grounded in real-world usage and challenges. The students who participated in the questionnaire surveys were drawn from the author’s own teaching context, comprising a diverse group of learners from different academic majors and language proficiency levels. These students were selected because they had been exposed to the locally developed materials as part of their English language learning journey.

Similarly, the teachers who participated in the interviews were colleagues of the first author and had been actively involved in the development and implementation of the locally produced ELT materials. Their first-hand involvement in the material development process, as well as their roles as instructors using these materials, made them valuable informants to provide deeper insights into the challenges and potential solutions.

The sampling procedure for both the questionnaire surveys and interviews followed a convenience sampling approach. Given the contextual nature of this study and the need to access participants directly involved in the use of locally developed ELT materials, this approach was deemed most appropriate. Convenience sampling allowed for the selection of participants who were readily accessible and willing to participate, ensuring a practical and efficient data collection process.

Furthermore, it should be noted that the participants in this study were selected based on their relevance to the research objectives, as they represented key stakeholders in the utilization and development of local ELT materials. Their perspectives and experiences were considered essential for addressing the research questions and providing valuable insights into the challenges and potential solutions related to these materials.

Overall, the selection of participants and the sampling procedure were carefully considered to align with the research focus, context, and objectives, ensuring that the insights gained from this study are meaningful and contextually relevant.

Initially, the authors conducted interviews with those teachers and students, to gain a deep understanding of their experiences and feelings. Details of interview participants are presented in Table 1. During the interview, the participants were informed that their responses would not be judged as right or wrong, and they were encouraged to employ anecdotes to substantiate their perspectives. The duration of each interview was approximately 30 min. Furthermore, the investigation delved into the attitudes of educators in habitual teaching practice, as well as the inclinations of students in their daily learning practice, by examining documents like teaching records and students’ lecture notes. All data were collected in Chinese to ensure the precise identification of the viewpoints of the interviewees. Two coders, the first author and the other, who are both proficient in qualitative research, conducted coding on interview transcripts. After reaching a consensus for each coding criterion, the coders completed the remaining coding task. Following the coding process, we calculated the interrater reliability to assess the consistency of coding between the two coders. The interrater agreement reached 93%, indicating a high level of agreement between the coders.

**Table 1:** The profile of teachers and students who participated in the interviews.

Identity	Teachers	Gender	Age	Title
Teacher	T1	Female	44	Lecturer
	T2	Female	38	Lecturer
	T3	Male	37	Associate professor
Identity	Students	Gender	Age	Academic year
Student	S1	Female	18	Freshman
	S2	Male	18	Freshman
	S3	Female	19	Freshman

Pseudonyms. Profile collected in the summer of 2022.

Subsequently, a questionnaire was distributed among the students to further collect relevant data pertaining to their attitudes towards the materials. The questionnaire used in this research was meticulously designed to align with the key themes identified during interviews. These themes encompassed language quality, content appropriateness, the integration of ideological and moral elements into materials, and multi-dimensional aspects of material development. The questionnaire items were crafted to capture participants’ perspectives and experiences related to these dimensions. To ensure the reliability of the questionnaire, several steps were taken. First, a pilot test of the questionnaire was conducted with a small group of participants who were not part of the main study. Their feedback and responses were analyzed to refine the wording and clarity of the questionnaire items. Subsequently, the finalized questionnaire was administered to a larger sample of participants, including both teachers and students, who were directly involved with locally developed ELT materials. The reliability of the questionnaire was further assessed using statistical measures, including Cronbach’s alpha coefficient, which yielded a high level of internal consistency for the questionnaire items. A total of 101 valid questionnaires were obtained from first-year students who were non-foreign-language majors. The respondents comprised 26 male and 75 female students. After conducting a reliability analysis on the questionnaire, the obtained α coefficient of 0.711 indicated that the questionnaire items were reliable in measuring the intended constructs. The quantitative data then underwent descriptive statistical analysis. In this research, our foremost commitment lies in upholding ethical standards and ensuring the trustworthiness of our investigation into locally developed ELT materials. We rigorously obtained informed consent from the teachers and students who participated, underscoring the principles of voluntary involvement and data confidentiality. Additionally, to enhance the reliability of our data, we employed triangulation, which amalgamated information from both interviews and questionnaires.

## 4. Findings

### 4.1. Overview

**Figure 1:**
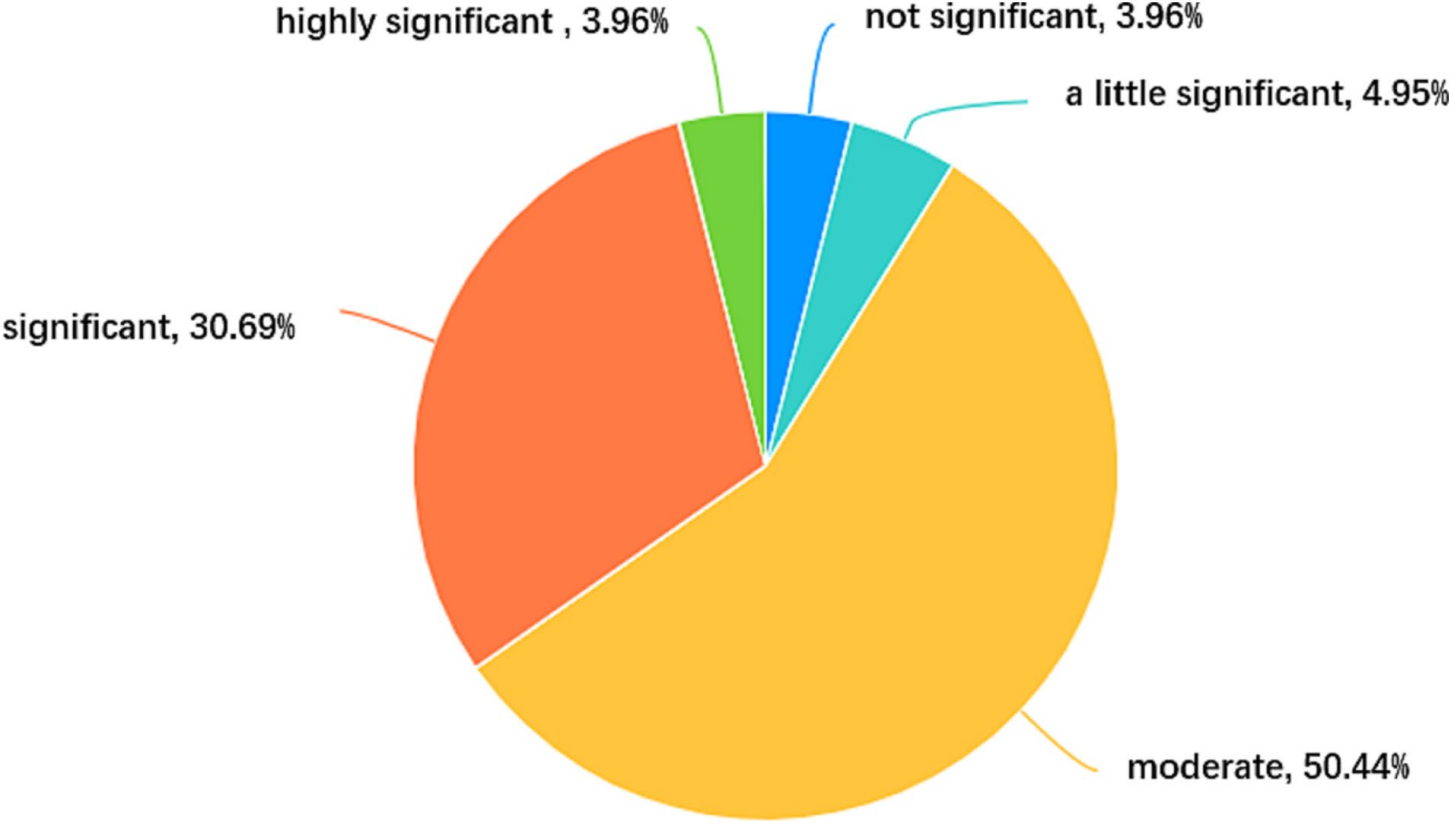
The impact of using the locally developed materials on students’ values.

The findings of this survey indicate that the ELT materials, which was locally developed based on the requirements of curriculum-based value education, have exerted a discernible positive influence on the advancement of students’ moral and ideological development, as illustrated in Figure 1. Over one-third (34.65%) of the students conveyed that the materials had a significant or highly significant impact on their values. Since the materials have been in use for less than a year, this ratio, to some extent, attests to their pedagogical effectiveness. However, the survey also unearthed several shortcomings encompassing the four aspects — language quality, content, the integration of local ideological and moral elements, and the multi-dimensional development of local ELT materials. These deficiencies will be further elaborated upon in the subsequent sections, drawing upon the insights garnered from the survey results, which will be elucidated upon in the subsequent sections, drawing upon the survey results.

### 4.2. Deficiencies

#### 4.2.1. Language

The investigation revealed that language in locally developed ELT materials in the context of curriculum-based value education is a significant issue that requires material developers’ attention. All surveyed teachers acknowledged that the language used in the texts and exercises in the set of ELT materials was insufficiently classic and authentic. For instance, T1 pointed out:

Extract 1

*The texts contain some awkward sentence structures, a phenomenon that I find quite prevalent in the materials. It is possible that in certain instances, non-native speakers use distinctive expressions to underscore a particular point, a practice that we can certainly tolerate. Nonetheless, I believe that frequent occurrences of such sentences in the texts are undesirable from a pedagogical standpoint. We should expose students to more classic expressions instead. (T1, interview)*

Teacher T3 expressed a similar view. She believed that it is crucial to use concise and authentic expression so as to tell Chinese stories well, while the language used to discuss Chinese issues in the locally developed ELT materials is verbose and difficult to comprehend. She also pointed out that the same problem occurred in the exercises of the textbook:

Extract 2

*The sentences provided in the textbook exercises are also very pertinent to ‘curriculum-based value education’, and the content expressed in them is lofty, but students cannot understand them. The sentences are too stiff, and there are too many new words. (T3, interview)*

The survey results indicate that only about 20% of students find the language in the locally developed ELT materials easy to understand. As for the causes of the difficulty in understanding the language used in the textbooks (Table 2), it mainly stems from aspects such as sentence length, sentence structure, and vocabulary. However, it is worth noting that 40.59% of surveyed students find that the understanding difficulty is increased by the values or principles that the sentences are trying to convey. Nearly one-third (27.72%) of students feel that some sentences are written by using uncommon expressions, and 18.81% of respondents believe that some sentences do not conform to idiomatic English expressions. These data are largely consistent with the opinions of the teachers, indicating that more attention should be paid to the language of the textbooks in the process of integrating elements of curriculum-based value education. Furthermore, 61.39% of students find that the language in the exercises is much more difficult than that in the texts. The design of exercise questions can be appropriately expanded based on the knowledge learned in the texts and the difficulty can be suitably increased, but overly complex sentences and too many new words may hinder students’ active exploration and even undermine their motivation to learn. As language materials, helping students make progress in “language” is of great importance. Neglecting the importance of the quality of “language” of the local materials will ultimately affect the effectiveness of curriculum-based value education in ELT.

**Table 2:** Students’ feedback on the language of the locally developed materials.

statements	The proportion of students who agree with this statement out of the total number (%)
the language in this series of locally developed ELT materials is difficult to understand	79.2%
the language in the exercises is much more difficult than that in the texts	61.39%
Some sentences tend to be lengthy	43.56%
Some sentences have rather complex structures	62.38%
Some sentences are not phrased idiomatically in English	18.81%
Some sentences use less common or peculiar expressions	27.72%
Some sentences have a high number of unfamiliar words	53.47%
Some sentences express ideas or concepts that are difficult to comprehend	40.59%
others	0.99%

The “language” problem may stem from the non-classical nature of the selected texts. As T2 suggested:

Extract 3

*After searching for articles featuring the authors’ names, I observed that these authors lack significant influence, and many articles are more likely excerpts from newspapers. In my opinion, we need more literary masterpieces, at least, less newspaper articles.*

Non-classic texts may not only pose problems in language quality but also affect the learning and interpretative value of the texts. The interviewed teachers all expressed their expectation for students to acquire genuine language skills through material-based learning, including the ability to emulate effective sentence structures and patterns to facilitate communicative and written activities. Nevertheless, if the language utilized in the text in the locally developed ELT materials is not sufficiently authentic, students will find it challenging to effectively carry out such activities.

One additional possible cause of the “language” problem is that there were no native speakers in the lists of developers or editors of these locally developed materials. T1 commented:

Extract 4

*I understand that Chinese editors can have a good understanding of the contents of the selected texts, but I still believe it would be advantageous to have native speakers engage in the editorial work to guarantee the quality of the language.*

#### 4.2.2. Content

The findings of this study disclose particular concerns relating to the content of the locally developed materials, particularly with respect to the complexity, selection of materials, and arrangement.

##### 4.2.2.1. The complexity

The text complexity can be evaluated based on several criteria, of which vocabulary constitutes a vital indicator (Read, 2000; Bulté and Housen, 2012). In this study, there was a shared consensus among teachers and students regarding the lack of stability in the vocabulary of the selected texts in the materials. For instance, Student S1 explicitly expressed the following opinion:

Extract 5

*I perceive that the level of difficulty in the textbook fluctuates, with certain units containing a conspicuous amount of unfamiliar vocabulary compared to others in the same volume.*

**Figure 2:**
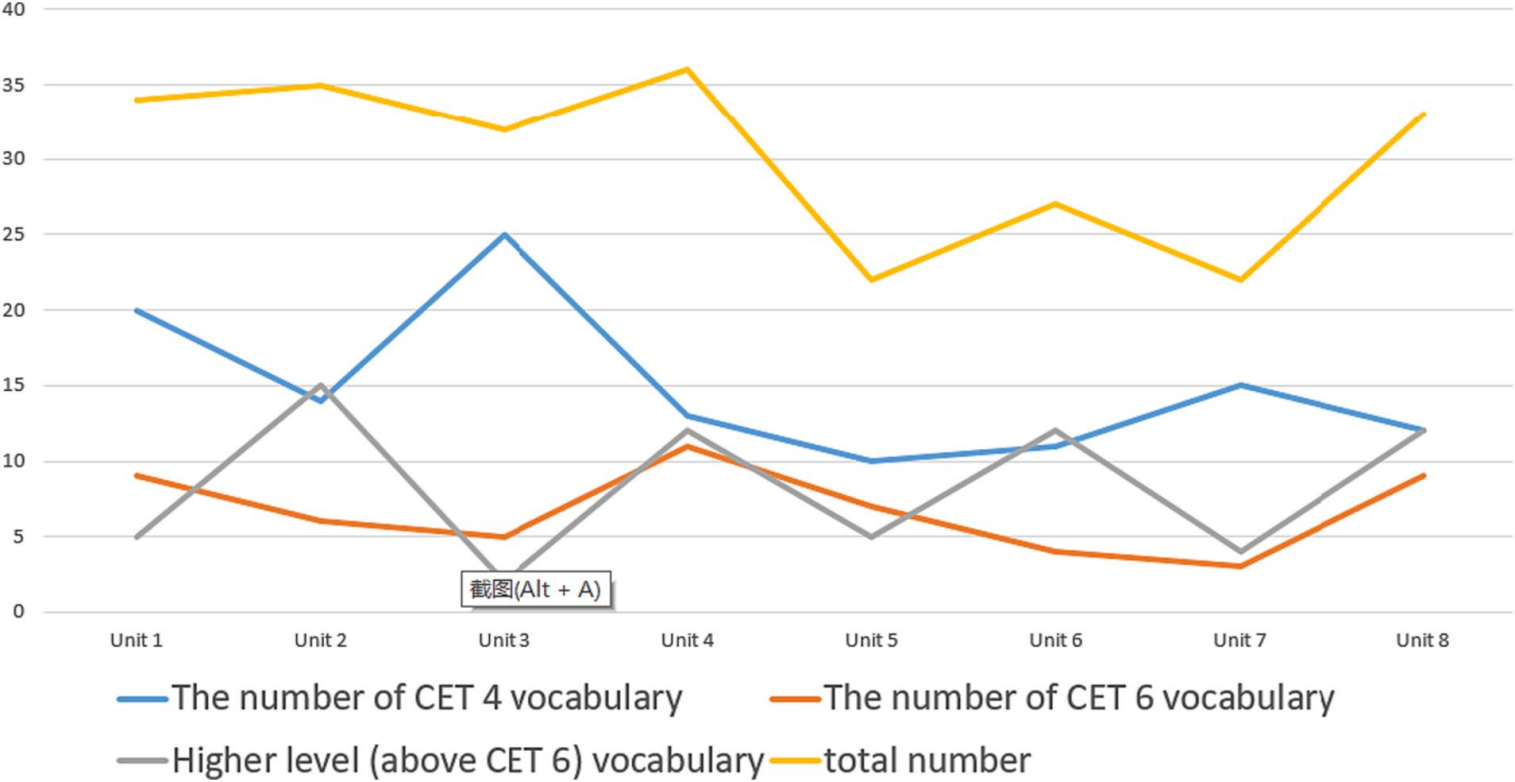
The distribution of vocabulary of each level in each unit of the locally developed materials (Take Volume 1).

To validate the assertion made by S1, the researchers utilized the vocabulary list appended to the textbook, and conducted a quantitative analysis of the distribution of College English Test Band 4 (CET-4), College English Test Band 6 (CET-6), and more advanced vocabulary across the eight units of the first volume of the textbook, as depicted in Figure 2. The results displayed a marked variability in the frequency of CET-4, CET-6, and more advanced vocabulary in the different units, revealing an insufficient consistency in the textbook’s lexical complexity. Therefore, it is necessary to implement certain modifications in this regard.

During the interview, T2 also shared her thoughts on the vocabulary:

Extract 6

*I believe the design of the vocabulary list could be more scientific. For example, the words drilled in the exercises are not completely consistent with that of the word list, which could affect students’ grasp of key vocabulary and their understanding of course content, thus compromising the effectiveness of the curriculum-based moral education*.

##### 4.2.2.2. Text selection

The selection of texts for locally developed ELT materials bears a direct correlation to the integration of ideological and moral elements in the context of curriculum-based moral education. Findings from this study revealed some problems in the process of selecting texts for locally developed materials. The first problem is that the selected texts were deemed too abstruse, and the content was perceived as not interesting and lacking in appeal to students. More than a quarter (26.73%) of the surveyed students (respondents of the questionnaire) expressed that the selected texts were relatively obscure and not germane to their daily lives. Additionally, almost four in ten (38.61%) respondents asserted that the materials’ content was not captivating enough. T3 mentioned that she and her colleagues agreed that the selected texts in the locally developed materials were somewhat tedious. She cited one of her colleague’s comment in the interview:

Extract 7

*I find this textbook difficult and boring. I tried to adapt the material and told my students from the beginning that the purpose of the materials is to evoke more reflection on certain values. In each lesson, I have been encouraging students to share their feelings on the topics in the textbooks by using examples from their own lives. However, the effects have been minimal as students find it challenging to do so as the contents in the selected texts are often very distant from their own lives.*

This demonstrates that when selecting texts for locally developed ELT materials, it is essential to not only ensure thematic relevance but also consider the suitability for the intended student audience. Given that the goal of the materials is to promote curriculum-based moral education, it would be prudent to conduct a thorough needs analysis among students when developing such materials. The needs analysis will facilitate the selection of texts and make the locally developed materials more likely to be well-received.

The second problem pertains to the inadequate coherence between the contents of the texts and the theme of the unit, which results in a lack of profound reflection on pertinent values. According to T2, some of the texts possess a seemingly substantial external presentation but offer little “substance” internally.

Extract 8

*For instance, there is an article on the topic of freedom where the author expresses a desire to be an ordinary researcher instead of a project leader or lab founder. However, the author’s accomplishments as a researcher are unclear. Upon my investigation, no remarkable achievements (of the author) were found.*

T2’s remarks suggest that there is no clear correlation between this text and the unit theme. Moreover, the author’s achievements are unremarkable, and the case concerned lacks adequacy and typicality, which renders it unconvincing. Such cases often fail to resonate with students and cannot play a good role in value guidance.

The third problem is that some of the texts presented in the locally developed materials depict an unfavorable image of China or reflect outdated images of China. For example, both interviewed teachers and students mentioned an article in which the author recounted being bitten by a spider as a child and how her mother treated the wound by using fire. Neither the teachers nor the students considered this text to be morally appropriate. T3 bluntly pointed out:

Extract 9

*The author uses this example to illustrate the backwardness of certain regions in China, and mentions at the end that achieving equal distribution of knowledge and education is necessary to eliminate such backward areas. But I think this plot is completely unnecessary. In this era, how many Chinese students still use fire to treat spider bites?*

S3 expresses her expectations for materials rich in values:

Extract 10

*I think the original intention of developing local ELT materials like this set of textbooks is to tell good stories about China, so there should be more positive examples. In fact, there are many such examples can be selected, which, I think, is easy to be found in Chinese culture.*

##### 4.2.2.3. Text arrangement

In the preface of the textbook, the authors underscored the significance of maintaining a rough equilibrium between Chinese and foreign cultural content. However, there appears to be no discernible arrangement pattern in this regard. According to our survey conducted among students, 66.34% of them found it challenging to determine whether the textbook achieved a balance between Chinese and foreign cultural content. T2 noted that while the first volume of the textbook usually features Reading A on foreign culture and Reading B on Chinese culture, this pattern is not consistently reflected in the second volume. T2’s perspective, which aligns with the expectations of both teachers and students, suggests that the authors should consider adopting a clear writing pattern to enhance students’ comprehension of the textbook’s content.

Additionally, T3 voiced her opinion on the organization of cultural contents in the materials based on the teaching practice. Given that college English courses now have fewer credit hours, T3 suggests:

Extract 11

*Chinese cultural contents should be presented in Reading A, as many universities tend to focus on learning this section, with Reading B only roughly covered or left for after-class study.*

While T3’s suggestion reveals that there is a need for local ELT materials developer’s to ensure a balance between Chinese and foreign cultural contents in each unit, and, if necessary, reduce the number of units. With the declining credit hours of the college English course, a well-structured arrangement in the ELT materials could prevent an imbalance in students’ exposure to Chinese and foreign culture.

#### 4.2.3. The integration approach of local ideological and moral elements

According to the notion of curriculum-based moral education, the concrete embodiment of “course-based ideological education” is preferred to be unobtrusive, and therefore the difficulty lies in how to integrate the ideological and moral elements into different courses (Shi and Wang, 2020). In interviews, both teachers and students expressed that this set of locally developed materials mainly present ideological and moral elements explicitly. Results from the questionnaire survey indicate that the vast majority of students felt like they were taking an ideological and moral course in English when using this set of materials (only 17.82% of students had no such feeling); a mere 14.85% of students preferred the explicit way of presenting ideological and moral elements in the materials. T1 also stated:

Extract 12

*The (ideological and moral) elements are not delivered subtly, I mean, in an implicit way. Instead, these elements are too obvious. It is somewhat difficult for students to get interested.*

It is apparent that this set of locally developed ELT materials have not yet to achieve the effect of being unobtrusive. The transition from explicitly presenting to implicitly integrating ideological and moral elements have not yet been realized. Clearly, to simply place ideological and moral elements in the texts, or to simply juxtapose different cultures there, is not the original intention of developing local ELT materials and cannot meet the needs of curriculum-based oral education.

It seems safe to say that whether the objectives of curriculum-based moral education can be achieved in an implicit way depends largely on teacher’s adaptations. Results from the questionnaire survey indicated that 37.62% of surveyed students reported that their teachers supplemented the locally developed materials with other resources in the teaching practice. In an interview, T1 mentioned that she added Chinese cultural elements to his lesson and tried to avoid preaching:

Extract 13

*For instance, when reading an article about nuclear leakage, which describes foreign scientists sacrificing themselves to protect others, I show a video clip of Chinese scientists to students and guide them in discussing what Chinese scientists have done, gradually inspiring everyone. The main approach is to guide students with something interesting, but I cannot say I excel in this practice.*

Teachers’ agency and their adaptations of the ELT materials are important in integrating ideological and moral elements into the course, but developing good local ELT materials is still something that cannot be replaced. To achieve better value guidance, local ELT material developers should improve the ways of integration and increase the implicit supply of ideological and moral elements, thereby offering better solutions to users of the materials.

#### 4.2.4. The development of multi-dimensional local ELT materials

The current study has unveiled notable room for enhancement in developing multi-dimensional local ELT materials. Despite the preface of the materials mentioning the reliance on information technology and claiming the integration of mobile learning platforms and teaching resources, both teachers and students perceived the extended resources provided by the material developers to be inadequate. The principal concerns articulated in the feedback from teachers and learners are presented as follows.

##### 4.2.4.1. The incomplete supporting materials

The accompanying reference materials for teaching and learning are indispensable for providing teachers with guidance and enhancing students’ academic achievements. Nevertheless, feedback from both teachers and students indicates that the supplementary reference resources corresponding to the locally developed textbooks fall short of meeting their teaching and learning requirements. For instance, T1 pointed out that:

Extract 14

*Certain crucial questions lack reference answers, and subjective questions lack any answers or basic guidance. While we advocate for students’ independent thinking and self-reflection, a value-driven set of teaching materials cannot fall short of guidance altogether.*

Additionally, T3 expressed the need to improve the quality of the reference translations in the teacher’s books.

Extract 15

*The reference translation of the texts offered in the teacher’s book is literal and reads like a machine translation. This is, of course, my subjective judgment. But I tell you that the reference translation is not good enough. I understand to express the local things in English is difficult and then translate these texts with local elements may be more challenging. However, I believe that ELT material development is a comprehensive undertaking, that is, apart from the formal textbooks, supporting materials such as reference translations must also be taken into account.*

The opinions expressed by the aforementioned teachers are quite representative. Besides teachers’ own endeavors, textbook developers should proactively offer “scaffolding” for teachers’ growth in their teaching competence in the new context of curriculum-based education. This includes providing appropriate guidance on some of the crucial or relatively covert value judgments that arise in the texts or exercises in the teaching supporting materials. In addition, students have also raised concerns about the related reference materials for the texts. S2 expressed his frustration:

Extract 16

*The WeChat mini program (that complements the content of the textbook) for vocabulary learning only offers word pronunciations and simple explanations, without any sentences or additional examples from the text. I stopped using it after a few times. Now I simply rely on the textbook and the materials provided by my teacher.*

S2’s comments indicate that local ELT material development should prioritize not only the form of multi-dimensional construction but also the quality of such construction. In the context of curriculum-based moral education, it is equally vital to pay attention to the inclusion of relevant ideological and moral education resources in the material packages. This approach ensures comprehensive presentation of ideological and moral elements and enables the multi-dimensional local ELT materials to truly enhance students’ learning experiences, thus accomplishing nuanced impacts on cultivating students.

##### 4.2.4.2. Insufficient extended digital resources

Besides the necessity for explanatory and guiding learning materials pertaining to the text’s content, the provision of extended digital learning resources related to the theme of the textbook content is also a crucial aspect of multi-dimensional construction for the locally developed ELT materials. Nonetheless, there are certain inadequacies with this series in this regard. Results from the questionnaire reveal that only 37.62% of the surveyed students endorse the digital learning resources provided by the material developers, implying that there is still significant room for improvement in this respect. After exploring the foreign language smart learning platform recommended by the material developers, it turns out that only the relevant electronic lesson plans (PowerPoint slides) for this series of materials were available, and there are no question banks or other resources accessible. Moreover, the digital courses on the learning platform require payment before they can be accessed. Users need to install the corresponding computer client to get access to the courses. However, when the author attempted to install the computer client, it was discovered that the client was no longer available for download. In response, T3 stated in the interview:

Extract 17

*I am currently teaching the second volume of the series this semester. The only resources available to me are the textbook, PowerPoint slides, and a teacher’s book. All other resources need be sought out by myself. It is quite time-consuming and laborious to locate English materials that are properly relevant or aligned with the content on core socialist values and Chinese social culture covered in the textbook, particularly when it comes to audio-visual materials, which are harder to find and require careful screening by myself (to ensure that the content conforms to the core values of socialism).*

The feedback from this teacher indicates that the materials have some deficiencies in terms of the form and content of the multi-dimensional resources. Teachers lack adequate materials that incorporate the ideological and moral elements as a leverage when conducting teaching and students lack them when engaging in extended learning. Hence, they have to compensate for this deficiency by their own efforts. While it is sensible for teachers to adapt the locally developed materials, the scarcity of multi-dimensional resources undoubtedly impairs the materials’ functionality. As an extension of the locally developed textbooks, digital supplementary resources are crucial for teachers to conduct effective curriculum-based moral education in ELT courses. The material developers need to offer practical and efficient support in this regard.

## 5. Solutions to the challenges

This study comprehensively investigates the actual use of a series of local ELT materials developed in the context of curriculum-based moral education in China and fully comprehend the feedback from material users (teachers and students) through questionnaires and interviews. Problems of the locally developed materials were identified, including the language quality, content appropriateness, integration of local ideological and moral elements, and the multi-dimensional construction of such materials. Based on these findings, this paper proposes the following solutions to optimize the development of local ELT materials.

### 5.1. Fundamental principle: Establishing a collaborative community for developing local ELT materials

The development of language teaching materials is a systematic project that requires the collaborative efforts of different entities (Tomlinson, 2011, p. 25). Establishing a cooperative community encompassing multiple stakeholders would be a significant approach to enhance the quality and effectiveness of locally developed ELT materials. This study reveals the importance of voices from material users: the concerns of teachers and the needs of students ought to be addressed. Their roles in the local ELT material development community should be accentuated. It is also important to consider the perspectives of researchers. Developing local materials is dissimilar to developing international materials; thus, research findings arising from local contexts are indispensable. Support from teacher educators and publishers is also required. Teacher educators can provide teachers with guidance on the scientific utilization of local ELT materials, and publishers can play a vital role in content review and other aspects. Material editors or textbook authors, naturally, occupy significant positions in the community. It is noteworthy that not only local authors but also native speakers (who are experts in material development) should be included in the community.

### 5.2. A framework for evaluating and improving locally development ELT materials

Drawing upon the findings of this study, we suggest a model for the evaluation of locally development ELT materials, as depicted in Table 3. The framework comprises four key aspects that should be taken into account when planning, developing, or using a series of local ELT materials: language, content, the integration approach of the local elements and the multi-dimensional construction of the locally developed materials. We assert that the items included within the framework would augment the quality of locally developed materials, not only in China but also in other parts of the world where such locally developed materials are needed for ELT.

**Table 3:** A framework for the evaluation and improvement of locally development ELT materials.

Dimension	Specific criteria that need to be considered
Language	Is the language used in the texts and exercises of the local ELT materials sufficiently authentic? Is the language used in the texts and exercises of the local ELT materials conforms to norms of English? Is the language used in the texts and exercises of the local ELT materials obscure and difficult to understand?
Content	Is the difficulty level of the local ELT materials appropriately controlled? Are the selected texts of the local ELT materials teachable? Are the selected texts the local ELT materials interesting and closely related to students’ daily life? Is the arrangement of local and global elements appropriate? (For example, whether the order is reasonable, or whether the constraints of the environment, e.g., the credit hours are considered) Are the incorporated local elements positive and constructive? Do the incorporated local elements have educational significance? Are the selected texts perfectly aligned with the unit theme?
The integration approach of local ideological and moral elements	Is the integration approach of local ideological and moral elements acceptable to the students?
Multi-dimensional development of Local ELT Materials	Are there sufficient supporting resources relevant to the teaching material content? Are there sufficient multi-dimensional databases/e-materials available as extension resources to expand the teaching and learning space?

Ensuring the quality of language in locally developed ELT materials is of primary significance. It necessitates an authentic representation of language, adherence to English language norms, and clarity in communication. Addressing the problem of language quality in ELT materials requires the joint efforts of both textbook editors and researchers. Firstly, it is suggested that English native speakers (who are experts in material development) should be included in the community of developing local ELT materials. They can play a vital role in reviewing the language used in the locally developed ELT materials, including texts, exercises, and supplementary materials, to ensure the readability and ease of learning. Secondly, the role of corpora in developing local ELT materials cannot be neglected (Tan, 2002, p. 5) to ensure the language quality of selected texts. Material editors can utilize corpora built by researchers to facilitate adherence to English language norms in local ELT materials, enabling students to authentically express and elaborate on local cultural and moral elements in English.

The findings of this study also suggest that developing locally developed ELT materials requires careful consideration of various content-related aspects. It involves controlling the difficulty level, ensuring teachability of selected texts, enhancing relevance to students’ daily lives, appropriate arrangement of local and global elements, and incorporation of positive and educationally significant local elements. Alignment with unit themes is also crucial for effective material development. To -improve the quality of content in locally developed ELT materials, several critical efforts should be undertaken. Firstly, Local material developing experts need to make efforts to incorporate local moral, cultural and ideological elements into the content. It should also be noted that it is crucial to highlight local culture and values in local ELT materials while simultaneously incorporating international and intercultural elements. Scientific organization and arrangement of these elements need to be carefully considered as well. Secondly, by conducting surveys on material users, factors such as learners’ age, major, daily life, cognitive characteristics, teachers’ teaching competence and teaching practice, and school credit hours should be considered; the users’ opinions on language quality, content appropriateness of the materials need to be fully considered and their needs for multi-dimensional local ELT materials should be addressed. Based on the survey results, material developers should aim to maintain a reasonable level of difficulty of the selected texts, and incorporate targeted, varied, interesting, and instructive local ELT materials that can enhance the appeal of the locally developed materials and increase learners’ motivation. In the context of this study, for example, it is recommended that the theoretical and practical research on curriculum-based moral education in ELT be further conducted and the results of such research be closely incorporated into the development of local ELT materials. A third possible solution is to construct a systematic and dynamic local ELT material database full of resources with clear local themes and positive local content, which may need collaborative efforts of policy makers, researchers, material developers and teachers. For instance, the resource database can be shared nationally with the help of policy makers and can be gradually enlarged by other material editors and teachers all over the country, which can make the development of local ELT materials more scientific.

The acceptability of the integration approach of local ideological and moral elements to the students is another pivotal factor in the development of locally produced ELT materials. As for how to incorporate local elements into ELT materials, continuous innovation is required in practice. For instance, in the context of this study, many material developers opted for manifesting ideological and moral aspects in an explicit way when developing local ELT materials based on the notion of curriculum-based moral education (Zhang and Wang, 2021). It is conducive to accentuate their value orientation by explicitly displaying some ideological and moral elements. Nevertheless, this explicit approach may engender students’ confusion about “whether I am taking a foreign language course or a moral education course” if persistently employed. Hence, material developers should endeavor to alter their writing mode that predominantly manifests local culture and moral aspects overtly. By taking teachers’ experience and feedback in using the materials into account and considering students’ knowledge level, practical needs, life experience and so on, they can integrate local cultural and moral elements into local ELT texts, exercises and other extension materials in a way that students are willing to accept, such as introducing specific cases, adapting the language, designing illustrations and other methods in the materials, and then achieve a real integration of local culture and moral education with language learning.

The development of locally produced ELT materials necessitates the availability of ample supporting resources relevant to the teaching material content, as well as an adequate supply of multi-dimensional databases and e-materials for expanding the teaching and learning space. With the continuous development of information technology in language education, the new generation of university ELT materials should break through the flat way of language learning, and the promote multi-dimensional construction of materials and realize the full extension and expansion of classroom learning through the deep integration with various information technology means and online learning platforms (Chen, 2007). Therefore, in the future, local ELT textbook development should gradually shift from textbook-centered writing to a new developing mode, that is, textbooks are only one important part of the rich multi-dimensional material resources. Especially with the continuous development of new artificial intelligence technologies such as ChatGPT and the hybrid teaching model in the post-epidemic era, local ELT textbook developers should focus on developing multi-dimensional digital resource databases and multi-dimensional teaching platforms as the key tasks to establish a smart local ELT material system and expand the local education space in ELT. On the one hand, local ELT textbook writers need pay more attention to the scale and modals of the moral elements of ELT materials. They should place emphasis on creating a multi-dimensional local ELT material system, with the multi-dimensional digital resource databases and the multi-dimensional teaching platforms serving as essential components. This will help to expand the space for the local education in ELT. On the one hand, in the current advocacy of “deep teaching” and “deep learning,” material developers should also pay attention to the “depth” of the multi-dimensional local ELT materials, and strive to realize the mutual complementation, promotion, and penetration of various modalities of local cultural, moral, and ideological education resources, so as to deepen students’ understanding of local elements and promote teachers’ deep teaching and students’ deep learning.

## 6. Conclusion

This paper empirically examines the challenges of developing local ELT materials from the micro perspective of material users, including both teachers and students. Drawing upon the concept of a material development community, this study has unearthed valuable insights into the challenges faced in the development and utilization of these materials.

One of the significant findings of this investigation pertains to language quality. It has been observed that the language used in local ELT materials often falls short in terms of authenticity and adherence to English language norms. This discovery underscores the importance of incorporating native English speakers, experts in material development, into the process to enhance language quality. Additionally, the utilization of corpora to maintain language standards in the materials has been suggested. Content-related aspects have also been a focal point of this study. The need for a careful balance in terms of content difficulty, teachability, and relevance to students’ daily lives has been emphasized. Furthermore, the strategic incorporation of local cultural, moral, and ideological elements alongside international and intercultural components has been highlighted. The organization and arrangement of these elements have been recognized as critical factors in the effective development of local ELT materials. Addressing the integration of local ideological and moral elements, this study underscores the importance of considering students’ acceptability of such integration. While explicit manifestation of these elements can be valuable, it must be done judiciously to avoid overwhelming students and ensure a balance between language and moral education. Moreover, in the quest for multi-dimensional development, the availability of ample supporting resources relevant to the teaching material content and the existence of comprehensive multi-dimensional databases and e-materials have been identified as pivotal. These resources are crucial for expanding the teaching and learning space, especially in the digital age.

In response to these findings, this study proposes a framework for evaluating local ELT materials, offering a systematic approach to material development that encompasses language quality, content appropriateness, the integration of local elements, and multi-dimensional construction. This framework aims to guide future material developers in creating effective and engaging materials aligned with the goals of curriculum-based value education.

Future research may involve the examination of a broader range of materials, a deeper exploration of various stakeholders’ attitudes toward material development, and the validation or augmentation of the findings presented in this study. By continuing to delve into these aspects, we can further refine the development and implementation of local ELT materials, ultimately enhancing English language education in the context of curriculum-based value education.

In essence, this research not only provides practical solutions for the enhancement of local ELT materials but also contributes to the broader discourse on material development in the evolving landscape of English language education.

## Data availability statement

The original contributions presented in the study are included in the article/supplementary material, further inquiries can be directed to the corresponding author.

## Author contributions

All authors listed have made a substantial, direct, and intellectual contribution to the work and approved it for publication.
